# Editorial: Spatial immune cell heterogeneity in the tumor microenvironment

**DOI:** 10.3389/fimmu.2024.1377532

**Published:** 2024-02-21

**Authors:** Anirban Ganguly, Sumit Mukherjee, Sheila Spada

**Affiliations:** ^1^Department of Biochemistry, All India Institute of Medical Sciences, Deoghar, Jharkhand, India; ^2^Actinium Pharmaceuticals, Inc., New York, NY, United States; ^3^Tumor Immunology and Immunotherapy Unit, IRCCS-Regina Elena National Cancer Institute, Rome, Italy

**Keywords:** immunotherapy, cancer, CAR-T, tumor microenvironment, oncoimmunology

In the era where immunotherapy is emerging as a pillar for cancer therapy, tumor heterogeneity represents a hidden barrier to clinical success of immuno-oncotherapy. The heterotypic interactions between cancer cells and the immune cells provide a complex dynamic platform that significantly impacts tumor progression and subsequent response to treatment (1, 2). The reciprocal interplay is influenced by local alterations, including different regional stiffness, co-existing vascularized and hypoxic areas, spatial genotype differences, and different distribution of immune cells. Cancer cells are capable of immune escape from both innate and adaptive immune cells (3). The immune checkpoints have certainly been novel therapeutic targets to generate robust anti-tumor immune responses and have emerged as a prominent choice of cancer therapy (4). Yet, the benefit of immune checkpoint inhibitors (ICIs) has been demonstrated only in a small subgroup of cancer patients (5). In solid tumors, one of the major reasons for not obtaining an optimal response from the ICIs has been ascribed to the spatial heterogeneity of the tumor microenvironment (TME), due to the remodeling of the intrinsic tissue architecture that dramatically affects the distribution of tumor-infiltrating immune cells. This spatial heterogeneity leads to varying degrees of cancer immune escape phenomena resulting in partial or no response to ICIs or acquired resistance (6, 7). Hence, analysis of the TME and identifying factors affecting TME heterogeneity provides a promising source to develop immunotherapy biomarkers and design strategies to overcome acquired resistance to therapeutic modalities in cancer patients (8, 9). The Research Topic “*Spatial Immune Cell Heterogeneity in the Tumor Microenvironment*” comprises one mini-review, two reviews, and three original research articles.

The fact, that immune cell heterogeneity resulting from differences in composition, localization, density, and functional state of the immune cells, significantly modulates the immunotherapy response in cancer was highlighted in the research article by Molina et al. where they studied the immune cell composition by immunohistochemistry methods on radical prostatectomy specimens obtained from two cohorts of patients. In the peritumor area, they found increased infiltration of CD209+ immature dendritic cells and CD163+ M2-type macrophages associated with late-onset adverse outcomes, necessitating more investigations in larger cohorts to confirm the results.

The effect of the spatial distribution and functional heterogeneity of different subsets of leukocytes in the human head and neck squamous cell carcinoma TME was reported in the research article by Netzer et al. who demonstrated that CD68^hi^ CD163^lo^ and CD68^hi^ CD163^hi^ are mainly localized close to the tumor sites, whereas CD68^lo^CD163^hi^ are prominently accumulated in the tumor stroma. CD68^hi^CD163^lo^ and CD68^hi^CD163^hi^ subsets were mainly macrophages expressing CD206 and little CD80. On the other hand, CD68^lo^CD163^hi^ cells were predominantly dendritic cells that expressed more CD80 and less CD206. The interaction of PD-L1/PD-1 occurs near the tumor nests where PD-L1^hi^ and PD-1^hi^ cells are predominantly gathered. Interestingly, high density of PD-L1^hi^CD68^hi^CD163^hi^ cells or PD-1^hi^ T cells in proximity to the tumor significantly correlated with improved survival.

The immune cell populations of the TME in clear cell, as well as papillary renal cell carcinoma (ccRCC and pRCC, respectively), have been investigated in the research article by Govindarajan et al., where imaging mass cytometry reveals distinct immunologic profiles. The proportion of CD4+ T cells between disease subtypes is higher in ccRCC than pRCC with no significant differences in CD68+ macrophage composition. Tumor immune escape mechanisms through P2X7R signaling have been discussed in the mini-review article by Sainz et al. where the complex role of P2X7R in mediating anti-tumor immunity has been highlighted. Tumors may skew P2X7R signaling to promote immune escape via mechanisms such as expression of non-pore functional variants of the P2X7R with a lower propensity for NICD (NAD-induced cell death) and AICD (ATP-induced cell death), enabling them to prevent cell death and harnessing P2X7R-mediated stimulation for proliferation and growth. Dysregulated P2X7R-signaling in tumors can be useful as alternative targets to modulate immunotherapeutic treatments of cancer patients.

The impact of mast cell-derived factors on colorectal cancer development, along with the prognostic significance of mast cells in patients during the tumor progression, have been discussed in the review article by Liu et al. They have reported that angiogenesis, lymphangiogenesis, and the cancer progression are significantly attributed to the mast cells and hence, they will provide future targets for novel cancer immunotherapy. The review article by Basak et al. highlights the role of M2-polarized tumor-associated macrophages (M2-TAM) in tumors. M2-TAM, pro-tumor cancer-associated immune cells, are known to dictate TME heterogeneity and enhance both TME immunosuppression and therapeutic resistance to ICIs and other conventional treatments in solid tumors. This review describes a possible cause for immunotherapy resistance in the context of the role of M2-TAM in the TME and reports rational approaches to disrupt the tumor–TAM interaction. This study addresses cancer therapy from the aspect of strategically targeting cancer cells, cancer stem cells, and TAMs simultaneously.

To enhance the effectiveness of immunotherapies and overcome therapy resistance, it is necessary to conduct a comprehensive study of spatial immune heterogeneity. This will improve the therapeutic decisions and lead to the discovery of more effective strategies for cancer treatment.

## Author contributions

AG: Writing – original draft, Writing – review & editing. SM: Writing – original draft, Writing – review & editing. SS: Writing – original draft, Writing – review & editing.
